# Nasal Powder Formulations: In-Vitro Characterisation of the Impact of Powders on Nasal Residence Time and Sensory Effects

**DOI:** 10.3390/pharmaceutics13030385

**Published:** 2021-03-13

**Authors:** Marie Trenkel, Regina Scherließ

**Affiliations:** Department of Pharmaceutics and Biopharmaceutics, Kiel University, 24118 Kiel, Germany; mtrenkel@pharmazie.uni-kiel.de

**Keywords:** nasal drug delivery, mucoadhesion, rheology, slug mucosal irritation assay

## Abstract

Nasal drug delivery is still primarily associated with locally-effective drugs, but next-generation products utilising the benefits of nasal administration—such as easy access to a relatively permeable mucosa, the presence of immunocompetent cells, and a direct route to the brain—are under investigation. Nasal powders offer the potential to improve the drugs’ effects by providing higher resistance against the mucociliary clearance, and thus prolonging the contact time of the drug with its target site. However, suitable and easy-to-use in-vitro setups tailored to the characterisation of this effect are missing. In this study, a selection of excipients for powder formulations were used to evaluate the applicability of different methods which investigate the influence on the contact time. The combination of the assessment of rheological properties, dynamic vapour sorption, and adhesiveness on agar–mucin plates was found to be a valuable predictive tool. For the additional assessment of the sensations associated with the close contact of powders and the mucosa, a slug mucosal irritation assay was conducted and adapted to powders. These methods are regarded as being especially useful for comparative screenings in early formulation development.

## 1. Introduction

Nasal drug administration is attractive, not only for the treatment of local diseases, but also for systemic delivery, the needle-free application of peptides, and the targeting of the central nervous system [1,2]. The rising awareness about nasal products will lead to a higher number of challenging drug candidates in product development, requiring formulations that are more sophisticated than the common liquid sprays or drops which strongly dominate the market nowadays. The first powder formulations that entered the marked were locally-acting products against hay fever (Teijin Rhinocort, beclomethasone dipropionate, Teijin, Japan in 1986 and Erizas, dexamethasone cipecilate, Nippon Shnyaku, Japan in 2012), and are commercially available in Japan. In 2016, Onzetra Xsail (sumatriptan; Currax Pharmaceuticals, Morristown, NJ, USA) was the first nasal powder formulation for systemic action approved by the food and drug administration (FDA), followed by Baqsimi (glucagon, Eli Lilly, Indianapolis, IN, USA) in 2019. These two approvals within the last five years may show a future trend, because powder formulations serve some unmet needs in nasal drug delivery. They show a higher stability, allow the administration of higher drug doses, and have been found to enhance the bioavailability in comparison to liquids [3,4,5,6,7,8].

A key challenge that can be addressed through the use of powder formulations is the limited residence time of drug substances in the nose due to mucociliary clearance [9,10]. As a physiological cleaning mechanism, the beating of the nasal cilia leads the upper gel-like mucus layer, which covers the epithelium, to move with a velocity of 6 mm/min towards the nasopharynx and throat [11]. Hence, drug particles from conventional liquid formulations, after being deposited on the mucus surface, are removed from the nasal cavity in about 15 min. Powder particles have shown higher resistance against the ciliary beat [8]. This effect may be tailored by the specific use of excipients. Useful excipients in nasal powder formulations would be soluble or insoluble fillers, mucoadhesive agents, or adsorption enhancers including enzyme inhibitors [3], among which mucoadhesive polymers appear to have the greatest potential to prolong the residence time in the nose. When polymer particles come into contact with nasal mucus, the polymer chains hydrate, while the surrounding mucosa dehydrates. Furthermore, hydrated polymers may entangle with the mucin of the mucus layer. The resulting close contact of the particles with the mucosa, specific interactions with mucin, and the change in mucus rheology prolong the nasal residence time.

However, suitable in-vitro setups for powders, tailored to the characterisation of this effect, are missing. While the applicable FDA guidance for industry explicitly does not include nasal powders [12], they are included into the guideline on the pharmaceutical quality of inhalation and nasal products of the European Medicines Agency (EMA) [13]. Both regulatory agencies focus in their recommendations on physical characterisation, the assessment of particle size distribution, the uniformity of the delivered dose, and stability issues. These tests are irremissible for the safe and effective use of nasal drug products, but are not sufficient for the development and screening of new and sophisticated formulations. Complementary experiments, which are useful for the evaluation of the behaviour of the formulation in the nasal cavity, are comprehensively summarised in [14]. However, when a reproducible screening method for the evaluation of powders is sought, most methods prove to have limited applicability. Models that use nasal tissues are often described [15,16], but inter-individual differences may limit the comparability of the obtained results. Because liquids are still more prominent in nasal drug delivery than powders, most experiments were mainly adjusted for the characterisation of liquids until now. When mucoadhesiveness is evaluated using rheological measurements, solutions of the ingredients are generally used [17,18]. Transferring such data to powders is difficult, because the process of swelling which would occur in the nose is not represented. Another method that is used to assess mucoadhesive potential is the evaluation of the dripping of a formulation onto a vertical positioned plate covered with simulated nasal secretions [19]. While this method generated reproducible results for liquids, and thus may be assumed to be suitable screening method, the setup may bring difficulties for powders, because dripping can only be observed after liquefaction, which requires an adequate surrounding that allows the hydration of the sample.

Hence, the aim of this study was to provide a convenient set of methods and to evaluate their applicability for powder formulations. Therefore, a selection of excipients, containing fillers and mucoadhesive agents with different qualities, was investigated with regard to their rheological behaviour in simulated nasal fluid, water vapour sorption, and adhesiveness on agar–mucin gels. The methods facilitate the screening and comparison of powder ingredients with regard to their suitability to affect nasal residence time. They can therefore be considered particularly valuable for formulation development.

A second point, which is often underestimated, is the evaluation of sensory effects coming along with nasal drug delivery [20]. Irritation, sneezing, itching, or pain caused by the contact of powder particles with the highly-sensitive mucosa are difficult to assess in animal studies or in vitro setups. Lenoir et al. provided a tool for the prediction of such sensations on human mucosa, which is the slug mucosa irritation (SMI) assay. A correlation was demonstrated between an increase in mucus production in slugs and an elevated incidence of stinging, itching, and burning sensations in humans [21]. However, the test was so far only used for liquid formulations, so the second aim of this study was to evaluate the SMI assay as predictive tool for nasal powders.

The availability of appropriate characterisation and screening capabilities will be key for the optimisation of nasal powder formulations in the future.

## 2. Materials and Methods

### 2.1. Materials

Nine different mucoadhesive polymers and four different fillers were selected for this study. Substances with different properties regarding their interaction potential with mucus (solubility, charge), were used to investigate the respective effect in nasal drug delivery.

As mucoadhesive agents, the neutral polymers hydroxypropyl methyl cellulose (HPMC 400 and 4000, Shin-Etsu, Chiyoda, Japan), hydroxypropyl cellulose (HPC (G)/(M)) and hydroxyethyl cellulose (HEC (G)/(M) (both: Ashland, Covington, KY, USA; G: declared viscosity of a 2% solution (25 °C) 150–400 mPas; M: declared viscosity of a 2% solution (25 °C) 4000–6500 mPas), the amphoteric and soluble chitosan derivative carboxymethyl chitosan (Heppe medical Chitosan GmbH, Halle, Germany) and the anionic polymers sodium carboxymethyl cellulose (CMC, Sigma-Aldrich, Saint Louis, MO, USA, medium viscosity) and low-methoxyl (LM) pectin (Herbstreith & Fox, Werder, Germany) were used. Mannitol (Pearlitol 160C, Roquette, Lestrem, France) and lactose (Inhalac 230, Meggle, Wasserburg am Inn, Germany) were selected as the soluble fillers, and microcrystalline cellulose (MCC, Vivapur 102, JRS Pharma, Rosenberg, Germany) and colloidal MCC (Vivapur MCG 811 P, JRS Pharma, Rosenberg, Germany) were used as the insoluble fillers. The selection criterion for all of the substances was non-toxicity. Therefore, excipients were chosen that are included in the inactive ingredient database of the FDA for nasal or respiratory use (HPMC, HEC, Pectin, CMC, mannitol, lactose, MCC), are used in marked products in Europe (HPC), or are reported to be non-toxic in the literature (chitosan and derivates [22,23]).

The porcine stomach mucin type II was purchased from Sigma-Aldrich (Saint Louis, MO, USA).

### 2.2. Preparation of the Powder Samples

For better comparison, sieve fractions of the raw materials were used in the experiments. In order to ensure successful deposition in the nose, particle sizes between 10 µm and 150 µm are needed in nasal drug delivery [24]; thus, analytical sieves with mesh sizes of 32 µm, 90 µm and 150 µm were used to classify the raw materials. The substances were sieved on a laboratory sieve shaker (Retsch GmbH & Co. KG, Germany), and fractions of 32–90 μm, 90–150 µm, and 32–150 µm were obtained. The characteristics of the excipients are summarised in Table 1.

### 2.3. Determination of the Particle Size Distribution

The particle size distribution was determined by laser diffraction using a HELOS laser diffractometer (Sympatec GmbH, Germany) equipped with a RODOS dispersing system. The particles were dispersed with a dispersing pressure of 3 bar. The data was analysed according to the Fraunhofer theory. All of the measurements were performed in triplicate. 

### 2.4. Evaluation of the Influence of the Powders on the Nasal Residence Time

#### 2.4.1. Rheological Testing

The rheological measurements were conducted using a plate-to-plate rheometer with a plate diameter of 40 mm (CVO 120 HRNF, Bohlin Instruments, Germany). The samples were prepared by adding 30 mg of sieved powder to 1.5 mL of simulated nasal fluid (SNF, Table 2) [25] and vortexed for 20 s. The dispersions were allowed to rest for 1 min, or for 15 min before measurement, in order to model the conditions directly after the application to the nose, and at the end of the residence time with a physiological clearance rate. The measurements were conducted at the nasal temperature of 32 °C [26].

The steady-shear viscosity was measured at a constant shear rate of 1 Hz, which is the effective shear rate in nasal mucus. This shear rate results from the directional movement of the cilia of the nasal epithelium, which has a beating frequency of 10 Hz [27]. 

The viscoelastic behaviour was assessed by oscillation measurements. The frequency-dependent elastic (G′) and viscous (G″) moduli were recorded at a constant deformation of 0.02, which lies in the linear viscoelastic region of all of the samples. The dissipation factor was calculated according to Equation (1):

tan δ = G″/G′.(1)

#### 2.4.2. Adhesiveness on Agar–Mucin Gels

**Figure 1:**
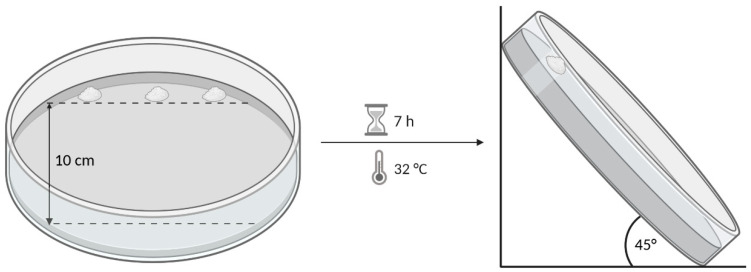
Schematic illustration of the experimental setup.

The mucoadhesive potential of the polymer samples was assessed by measuring the displacement of powder on agar–mucin and pure agar gels on an inclined plane. The method was adapted from Bertram and Bodmeier [28]. A hot solution of 1.5% agar with or without 2% mucin in phosphate buffer, pH 6.4 (Ph. Eur.), was cast on a petri dish (diameter 14 cm) and left for gelation in a refrigerator overnight. Prior to the test, the gels were stored for 1 h at a temperature of 32 °C in order to equilibrate to the test conditions. In total, 25 mg of powder (sieve fraction 32–150 µm) was placed on top of the gel in a spot with a diameter of approximately 10 mm. In order to start the test, the petri dishes were placed in an upright position with an angle of 45°, and the displacement of the powder samples was measured as a function of time. In order to assess the interaction of the mucoadhesive polymers with mucin, the test was conducted on agar–mucin and pure agar gels, and the difference in displacement was evaluated. The experimental setup is shown in Figure 1. All of the measurements were conducted in triplicate. The maximal measurable displacement on the plates was 10 cm. The displacements that exceeded this mark are listed as >10 cm.

#### 2.4.3. Dynamic Vapour Sorption (DVS)

The water vapour sorption of the substances (sieve fraction 32–150 µm) was determined using a DVS Resolution (Surface Measurement Systems Ltd., London, UK). The relative humidity was increased and decreased stepwise from 0% to 90% to 0% in isothermal conditions (25 °C). This program was conducted twice. Each step was held until mass equilibrium was reached. Hygroscopicity can be assessed by using the criteria from Ph. Eur. 10.0/5.11.00.00, which rate the gain in mass at 80% relative humidity and 25 °C. A mass gain of 0.2–2% is classified as slightly hygroscopic, a mass gain of 2–15% is classified as hygroscopic, and a mass gain of 15% and higher is classified as very hygroscopic.

### 2.5. Evaluation of the Sensory Effects in the Nose

#### Slug Mucosal Irritation Assay

In order to assess the potential of the powder samples for stinging, itching, and burning sensations in humans, the method of Lenoir et al. was adapted and tested for powder formulations [29]. The test is based on the assessment of the amount of mucus produced by slugs of the species Arion lusitanicus, which are placed in contact with the sample, as a measure for the extent of the sensory effects in the nose. In [21], a correlation between the nasal discomfort, reported by participants of a human nose irritation test after the use of liquid formulations, and the mean total mucus production of the slugs was found. Hence, the assay may serve as surrogate for clinical trials in the screening of nasal formulations. 

The slugs for the experiments were obtained by wild harvesting, and were kept under laboratory conditions. Two days prior to the experiment, slugs with a body weight between 3 g and 6 g were isolated (placed on paper towels moistened with phosphate buffered saline pH 7.4, PBS, Table 3) and their body wall was daily wetted with 1 mL of PBS and checked for any mucosal damages.

The samples were prepared and placed into petri dishes. As described in [29], 100 µL PBS was used as the negative control (no irritation) and 100 µL of a 1% (*w*/*v*) benzalkonium chloride solution was used as positive control (maximum irritation). Because PBS, as the negative control, leads to a weight gain of slugs due to hydration, the test was also conducted without sample as a negative control, because hydration can only occur with liquid but not with powder samples. The sample amount for the powders was adapted to 50 mg in order to display a proper dose for nasal powder administration. For all of the powder samples, a sieve fraction of 32 µm–150 µm was used. For the mannitol, sieve fractions of 32 µm–90 µm and 90 µm–50 µm were tested additionally, in order to investigate the influence of particle size.

At the beginning of the experiment, the slugs and the petri dishes containing the samples were weighed. Subsequently, the slugs were placed on the test substances for a contact period (CP) of 15 min. After the contact period, the slugs were transferred to petri dishes with 1.5 mL PBS for a resting period of 60 min, and the petri dishes containing the test substances and the produced mucus of the slugs were re-weighed. This procedure was repeated two times, so that a total mucus production out of three contact periods could be calculated. The results were displayed as the total mucus production (TM) in percent of the initial body weight (BW) of the slugs, according to Equation (2):

(2) $$\mathrm{TM},\;\%\;=\;\frac{1}{n}\overset{n}{\underset{i=1}{\sum }}M{\left(\mathrm{Mucus}\;\mathrm{per}\;\mathrm{CP},\;g\right)}_{i}/\;\mathrm{BW},\;g\;\text{×}\;100\%$$

All of the experiments were conducted with three slugs that were not used in any experiments before.

## 3. Results and Discussion

### 3.1. Evaluation of the Influence of Powders on Nasal Residence Time

**Figure 2:**
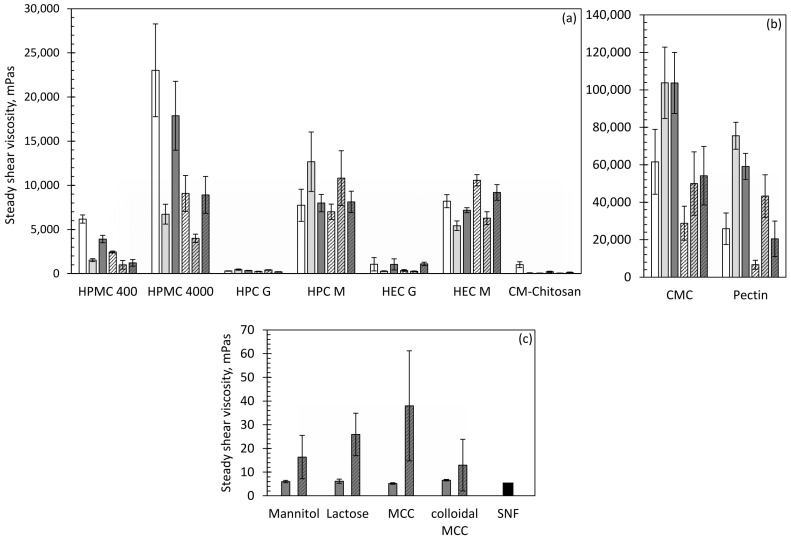
(**a**, **b**) Particle size-dependent steady shear viscosity at 1 Hz of the mucoadhesive polymers (2% in SNF). The white bars represent the sieve fraction (SF) 32–90 µm: light grey bars, SF 90–150 µm; dark grey bars, SF 32–150 µm. For better visibility, the data is presented in two graphs with different scaling of the y-axis. (**c**) Steady shear viscosity at 1 Hz of the fillers (SF 32–150 µm). (**a**–**c**) The plain bars show the steady shear viscosity after 1 min, and the striped bars after 15 min. *n* = 3; error bars = sd.

Because mucociliary clearance is strongly affected by the viscoelastic properties of nasal secretions, rheological assessments are used to evaluate the influence of the powders on nasal residence time. Mucus is typically considered to be a viscoelastic fluid [27]. As such, viscosity enables nasal mucus to sufficiently support a load, while elasticity restores mucus to its original shape after deformation by the cilia. Elasticity was found to be the most important for an efficient transport rate with an optimal elastic modulus of 1–2 Pa [30]. With a too-high elasticity, the mucus does not flow, while with a too-low elasticity, the mucus does not move like a continuous sheet [27]. The selected mucoadhesive polymers are expected to build up viscoelastic gels when they are in contact with the nasal fluid, and thus to reduce the mucociliary clearance [10]. The selected fillers are not expected to significantly increase the viscosity of SNF except for colloidal MCC, which is able to build up colloidal gels after adequate dispersion through shearing. The steady shear viscosity of the samples after 1 min and 15 min at a shear rate of 1 Hz is compared in Figure 2.

The two time points were assessed in order to simulate the change in viscosity from the first contact of the sample with the moisture in the nose to the end of the residence time at a physiological clearance rate. For the samples that show good dispersion and rapid dissolution in SNF, a further increase of the initial viscosity with the progress of time and swelling was expected, while for samples that are difficult to disperse in SNF, the solid particles may lead to a high initial viscosity, which decreases when the gels become more homogeneous. None of the samples showed a high increase of viscosity with the progress of time; indeed, for HPMC, CMC and pectin, a decrease in viscosity was observed. As observed visually, those samples rapidly build up highly-viscous areas around the polymer particles, which influenced the measurements after 1 min, while after 15 min the gels were more homogeneous. A rapid onset of gelling is required for nasal formulations to reduce the mucociliary clearance rate directly after administration. Moreover, a rapid increase of viscosity fixes the formulation to the original deposition site, which is required for targeted drug delivery [31].

The measurement of the steady shear viscosity was further used to quickly evaluate the influence of the particle size on the viscous behaviour of the polymer samples. On that point, no general statement can be made. While HPMC, HEC, and carboxymethyl chitosan show higher viscosity with smaller particles from 32–90 µm, HPC, CMC, and pectin show higher values with the larger particles from 90–150 µm. Hence, a broader particle size range from 32–150 µm was selected for the further experiments, in order to enable better comparability. The viscosity measurements of the fillers met the expectations for mannitol, lactose and MCC. For the colloidal MCC, it was not possible to build up a colloidal gel. Dissolved salts, such as those present in SNF, can inhibit the dispersion of colloidal MCC, which contains an anionic component, and therefore prevent gelling [32]. In this regard, colloidal MCC is not advantageous over MCC in nasal powder delivery, because a sufficient dispersion in the nose is not possible.

**Figure 3:**
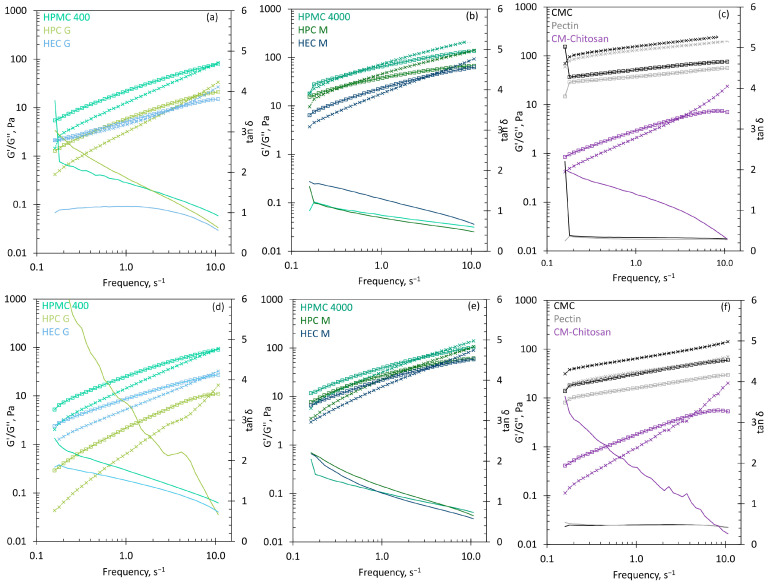
Frequency-dependent viscosity measurements of the mucoadhesive excipients: dotted lines with cross, elastic modulus (G′); solid lines with squares, viscous modulus (G′′); solid lines, dissipation factor (tan δ). (**a**) HPMC 400, HPC G, HEC G after 1 min resting time; (**b**) HPMC 4000, HPC M, HEC M after 1 min resting time; (**c**) CMC, Pectin, CM-Chitosan after 1 min resting time; (**d**) HPMC 400, HPC G, HEC G after 15 min resting time; (**e**) HPMC 4000, HPC M, HEC M after 15 min resting time; (**f**) CMC, Pectin, CM-Chitosan after 15 min resting time. The mean of the measurement of the three gels is shown; sd is not given for the clarity of the graph.

Oscillatory, strain-controlled measurements were conducted in order to assess the viscoelastic properties of the gels formed by the mucoadhesive polymers after 1 min and 15 min in SNF. The frequency-dependent elastic moduli (G′), viscous moduli (G″), and the dissipation factors, which express the ratio between the viscous and the elastic part of the viscoelastic deformation behaviour, are pictured in Figure 3. In oscillatory frequency tests, the time-dependent deformation behaviour is examined, with frequency as the inverse value of time. The short-term behaviour is simulated using rapid movements (i.e. high frequencies), and the long-term behaviour is simulated with slow movements (i.e., low frequencies).

The anionic polymers CMC and pectin show a gel character (G′ > G″) over the whole frequency range. Both materials immediately formed highly-viscous areas around the particles, which still existed after 15 min. The high values of the elastic modulus after 1 min indicate that mucociliary clearance would be strongly slowed down after the first contact of the powder with nasal secretions, and that the formulation may stick to its original site of deposition. The neutral polymers (HPMC, HPC, HEC) showed sol character at low frequencies and gel character at higher frequencies. The point of intersection (G′ = G″) occurred later for the shorter chain polymers (HPMC 400, HPC G, HEC G) than with the longer chain polymers (HPMC 4000, HPC M, HEC M). The longer chains are inflexible when they move rapidly, and are more likely to get entangled. Because interpenetration and entanglement are assumed to be crucial steps in the consolidation state of mucoadhesion [33], a high tendency to entanglement may be regarded as advantageous for excipients for nasal powder formulations. Carboxymethyl chitosan behaved similarly to the shorter-chain neutral polymers, with relatively low values for the elastic and viscous moduli. All of the polymers formed gels after one minute in SNF that had values greater than 1 Pa for the elastic modulus at 1 Hz. Hence, it can be assumed that all of the polymers will slow down the mucociliary clearance rate directly after administration to the nasal cavity. After 15 min, the viscous moduli became relatively higher, which is reflected in the higher values of the dissipation factor, and indicates that the effect on the mucociliary clearance rate will decrease with time. After the drug is absorbed, a recovery of the physiological clearance rate is needed to clear the insoluble components of the formulation and to regain the physiological functionality of the nose; thus, a time-limited effect is desirable. The duration of the effect may be controlled by the choice of polymer, and thus a sufficient extension of nasal residence time for the drug absorption can be ensured.

**Figure 4:**
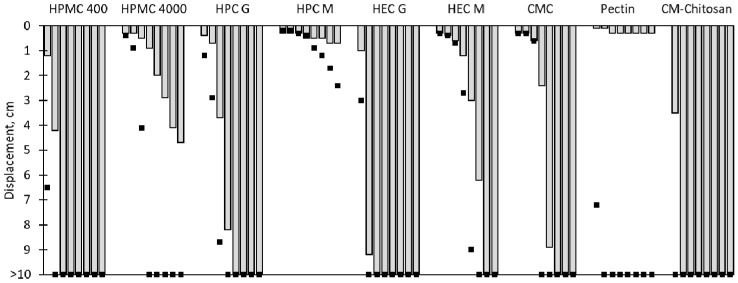
Displacement of powder samples on agar–mucin gels (bars) and pure agar gels (dots) after 0.5, 1, 2, 3, 4, 5, 6, and 7 h (from left to right). *n* = 3; the maximal displacement is shown.

Because the measuring of rheological properties is a rather artificial method which does not mimic the actual conditions in the nose, in which the dry particles are deposited on the surface of a moist mucosa, it is advisable to carry out a complementary method for the characterisation of this contact stage. When dry polymer particles come into contact with nasal mucosa, the polymer chains hydrate, while the surrounding mucosa dehydrates [34]. In order to reflect this wetting, the adhesion of dry polymer powders on agar–mucin gels and pure agar gels was assessed. On pure agar gels, the extent and velocity of the hydration can be examined. An increased adhesion on gels containing mucin indicates specific interactions between the sample and the mucin that contribute to its mucoadhesive properties. Figure 4 shows the displacement of the samples on an inclined plane of the gels.

Focusing on the neutral polymers, it can be seen that the shorter chain polymers moved expectedly faster than the longer chain polymers because of their lower viscosity after wetting, and because of a minor tendency to entangle. HPC dislocated the slowest on agar and ager-mucin gels of all of the neutral polymers, although the HPC gels did not show the highest viscous and elastic moduli in the rheological assessments. A potential reason for the earlier failure of the adhesive bonds of the other polymers is overhydration [33]. In order to substantiate this theory, the dynamic vapour sorption of the samples was recorded. It was found that HPC absorbed water vapour to a lesser extent than the other polymers, which may result in more highly concentrated, and therefore more stable gels on the agar plates. The results of the DVS measurements are summarised in Table 4. Carboxymethyl chitosan showed a rapid displacement, comparable with the shorter chain neutral polymers, which fits to its rheological properties. The displacement of the anionic polymers CMC and pectin, however, could not be predicted from their viscoelastic properties. While CMC showed little movement in the first two hours, and a later rapid displacement on both agar and agar–mucin gels, pectin showed very rapid displacement on the agar gels, but almost none on the agar–mucin gels. This result indicates a strong interaction of pectin with the used mucin type II. The interactions of different types of pectin with mucin were investigated in [35], and a high ability, especially of low methoxylated pectin, to engage in hydrogen bonding with mucin was assumed. The relatively-rapid displacement of pectin on agar gels, and of CMC, can be interpreted as adhesive failure because of overhydration due to the very hygroscopic behaviour of the powders (see Table 4).

Considering all of the results, the combination of the three methods was found to be suitable for a screening of the excipients with regard on their decelerating effect on the mucociliary clearance rate. Such an easy-to-use in vitro setup will be especially useful as a screening tool in the phase of the development and optimisation of a formulation. Other models, which are described in the literature for that purpose, are based on the use of nasal tissues, such as rabbit, sheep, goat, calf, and pig mucosa [14]. In [15], a wash-off technique is described, in which the formulation is spread onto the tissue and the amount that remained on the tissue after a defined washing circle was quantified. Another approach measures the required force to separate the formulation from the tissue after a defined period of intimate contact [16]. Because the stickiness of a formulation on nasal tissue does not necessarily match with its influence on ciliary movements, it cannot be correlated with its effect on clearance in vivo. Hence, these methods are also mainly useable as screening tools. However, tissues underlie interindividual differences, affecting reproducibility. This bias may be propagated if the sample cannot be applied to the tissue in a repeatable manner. When the tensile strength of adhesive bonds is assessed, a further problem lies in the evaluation of the obtained results, because fracture can occur not only at the interface between the mucosa and the sample but also within the sample or the mucus layer [33]. Therefore, methods that enable a higher degree of standardisation, such as the ones described in this study, are better suited for screening and quality testing.

The characteristics of mucoadhesive excipients will influence the residence time in the nose to different extents, and they will also affect the absorption of active ingredients (API) because hydrocolloidal matrices are formed upon the excipient hydration that the API has to pass before absorption. In [5], the use of different types of HPC were found to be beneficial for sumatriptan as a model drug with a high solubility and low permeability, but not for warfarin, as model drug with an already-high permeability. Regarding that, a conclusive statement of the advantageousness of mucoadhesive excipients needs to consider API properties. Therefore, in later stages of product development, further investigations such as cell culture and in vivo experiments are inevitable. Absorption studies need to be conducted in order to adjust and ensure the desired drug effect. Additionally, toxicity assays are required in order to guaranty the safety of the formulation.

### 3.2. Evaluation of the Sensory Effects in the Nose

**Figure 5:**
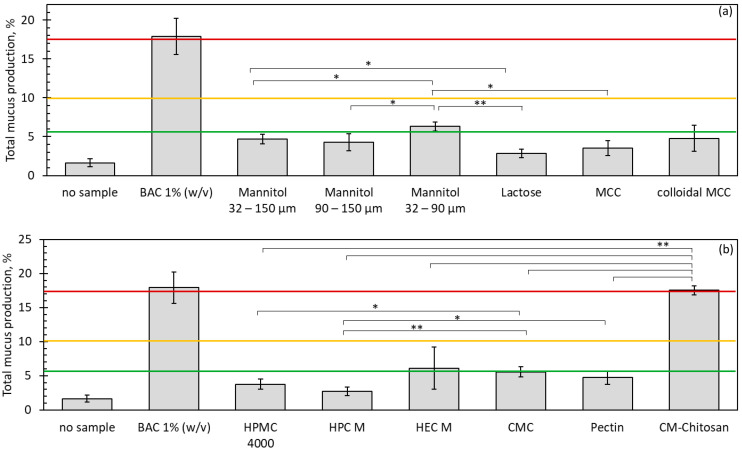
Total mucus production from three contact periods in the slug mucosal irritation assay, expressed as a percentage of the initial bodyweight of the slugs. (**a**) Fillers; (**b**) mucoadhesive polymers. Unless otherwise stated, sieve fractions from 32–150 µm were used. The lines display the limit values for the different categories of nasal discomfort, as defined in [21]: below the green line, no discomfort; below the yellow line, mild discomfort; below the red line, moderate discomfort; above the red line, severe discomfort. *n* = 3, error bars = sd, * = *p* < 0.05, ** = *p* < 0.01.

As described earlier, drug delivery via the nose requires the close contact of the particles with the nasal tissue. This may cause irritation on the sensitive mucosa. The slug mucosal irritation assay was used to screen the selected fillers and mucoadhesive agents regarding their potential to cause stinging, itching and burning sensations. A correlation between an increase in mucus production in slugs and an elevated incidence of these sensations in humans was demonstrated in [21]; thus, the assay may serve as a surrogate for clinical trials in early formulation development. The correlating mucus production of slugs that were in contact with the powders is displayed in Figure 5.

All of the fillers significantly (*p* < 0.05) increased the mucus production of the slugs. However, with the exception of mannitol with a small particle size, they are all within a range which is not associated with discomfort, according to [21]. Comparing the fillers, a significantly higher mucus production was observed in slugs placed on mannitol than on lactose. A potential reason would be the higher osmotic pressure that is caused by dissolved mannitol particles. When soluble powder particles come into contact with moisture, like the wet body wall of the slugs or the human nasal mucosa, increasingly concentrated solutions are formed over time, which is accompanied by the increasing osmolarity of the respective fluid. Because mannitol dissolves faster than lactose and has a lower molecular weight, dissolving mannitol particles increase the osmolarity faster. Hyperosmolar solutions will cause an efflux of water from the surrounding cells. Different studies already describe an increased tendency for nasal irritation for hyperosmolar saline solutions when compared with isotonic solutions, which is consistent with this theory [36,37]. However, the mucus production observed for mannitol is not significantly higher than for the selected insoluble fillers with the same particle size range. The influence of particle size was evaluated for mannitol, and a significantly higher mucus production was found for the sieve fraction 32–90 µm than for the sieve fractions 32–150 µm or 90–150 µm. The obtained total mucus production of 6.30 ± 0.61% of the initial body weight of the slugs indicates mild irritation in the nose, possibly due to a faster dissolution of the smaller particles and hence a more pronounced osmotic effect. Comparing the mucoadhesive excipients, carboxymethyl chitosan stood out with a high total mucus production of 17.52 ± 0.63%, which is associated with severe nasal discomfort, and ranges only slightly below benzalkonium chloride (BAC) 1% solution as positive control. The slugs tended to produce less mucus when they were contact with the neutral polymers HPMC and HPC than when they were placed on powders of the anionic polymers CMC and pectin. Contrary to this trend, the mucus production was slightly higher in HEC, but the standard deviation was high, so there was no statistically significant difference. For all of the other samples, results with quite small variations were obtained, allowing a meaningful comparison of the different excipients. Hence, the slug mucosal irritation assay is also a useful tool for the characterisation of powder formulations, and allows an early screening of formulations and excipients. Such results are often difficult to obtain otherwise, because clinical trials are costly, and are often used only at a later stage of development.

## 4. Conclusions

This work delivers a set of methods suitable for the assessment of excipients for nasal powders and nasal powder formulations. Adhesiveness on agar–mucin plates, rheological tests, and DVS measurements facilitate the meaningful characterisation of the samples, and a combination of these methods is regarded as being valuable for the prediction of their ability to enhance the residence time in the nasal cavity. Different mucoadhesive agents were compared using the described methods. Assessing the rheological tests, all of the samples are able to prolong the nasal residence time by increasing the elastic modulus at 1 Hz above 1 Pa. Longer polymer chains and an anionic charge were found to increase the effect. HPC and pectin showed the strongest adhesion on agar–mucin gels, which can be explained by a smaller extent of vapour sorption for HPC and specific interactions with mucin for pectin. However, the attributes of the active ingredient have to be considered in the interpretation of the data, and further investigations like cell culture and in vivo experiments, focusing on the estimation of the drug absorption on the one hand and on the evaluation of toxicity on the other hand, need to be conducted in the later phases of product development. In order to deliver a tool to predict the sensory effects of nasal powders, which come along with the close contact of particles and the mucosa, the slug mucosal irritation assay was adopted for dry powders. The test permitted us to differentiate between the samples with high reproducibility, and is therefore considered a suitable and useful screening tool in nasal powder development. The screening of the different selected fillers and mucoadhesive agents suggests the dependence of the nasal discomfort on the particle size and the charge of the powder components.

## Figures and Tables

**Table 1:** Characteristics of the excipients selected for this study.

Mucoadhesive Agent	Viscosity Grade	Charge	x_50_ of Sieve Fractions ± SD, µm		
			32–90 µm	90–150 µm	32–150 µm
HPMC (hydroxypropyl methyl cellulose)	400 (2%, 20 °C)	neutral	72.6 ± 1.3	141.6 ± 0.8	99.7 ± 1.6
	4000 (2%, 20 °C)		74.8 ± 1.0	139.5 ± 0.5	86.5 ± 0.7
HPC (hydroxypropyl cellulose)	G *		96.6 ± 1.0	179.8 ± 14.7	136.8 ± 9.2
	M **		92.1 ± 0.4	163.1 ± 0.7	131.4 ± 6.9
HEC (hydroxyethyl cellulose)	G *		64.7 ± 0.1	134.6 ± 1.5	78.9 ± 0.6
	M **		73.4 ± 1.6	140.3 ± 2.8	88.7 ± 0.5
CMC (carboxymethyl cellulose)	medium	anionic	74.6 ± 0.5	138.9 ± 4.4	95. 2 ± 1.9
Pectin	N/A		78.4 ± 0.6	133.0 ± 1.1	95.8 ± 5.4
Carboxymethyl Chitosan	N/A	amphoteric	65.8 ± 1.9	162.4 ± 1.1	142.4 ± 2.4
**Filler**		**Solubility in Water**	**x_50_ of Sieve Fractions ± SD [µm]**		
			**32–90 µm**	**90–150 µm**	**32–150 µm**
Mannitol		soluble	65.4 ± 0.1	160.3 ± 0.4	113.5 ± 2.0
Lactose			N/A	N/A	100.8 ± 0.5
MCC (microcrystalline cellulose)		insoluble	N/A	N/A	88.0 ± 0.5
colloidal MCC			N/A	N/A	70.7 ± 2.6

* G: declared viscosity of a 2% solution (25 °C) 150–400 mPas. ** M: declared viscosity of a 2% solution (25 °C) 4000–6500 mPas.

**Table 2:** Composition of simulated nasal fluid (SNF).

Ingredient	Concentration	pH
NaCl	7.45 g/L	6.4
KCl	1.29 g/L	
CaCl_2_ × 2 H_2_O	0.32 g/L	
double-distilled water	q.s.	

**Table 3:** Composition of phosphate buffered saline (PBS).

Ingredient	Concentration	pH	Osmolality
NaCl	8.0 g/L	7.4	288 mosmol/kg
KCl	0.2 g/L		
Na_2_HPO_4_	1.42 g/L		
KH_2_PO_4_	0.27 g/L		
double-distilled water	q.s.		

**Table 4:** Dynamic vapor sorption: change in the mass of the polymers at 80% relative humidity in the first cycle (*n* = 1).

Mucoadhesive Polymer	Change in Mass—Ref, % at 80% P/P_0_	Hygroscopicity
HPMC 400	13.84	hygroscopic
HPMC 4000	14.14	hygroscopic
HPC G	12.17	hygroscopic
HPC M	12.09	hygroscopic
HEC G	25.87	very hygroscopic
HEC M	25.36	very hygroscopic
CMC	31.13	very hygroscopic
Pectin	23.42	very hygroscopic
Carboxymethyl Chitosan	97.70	deliquescent

## Data Availability

Not applicable.
