# Correction: Trenkel, M.; Scherließ, R. Nasal Powder Formulations: In-Vitro Characterisation of the Impact of Powders on Nasal Residence Time and Sensory Effects. *Pharmaceutics* 2021, *13*, 385

**DOI:** 10.3390/pharmaceutics15071852

**Published:** 2023-06-30

**Authors:** Marie Trenkel, Regina Scherließ

**Affiliations:** Department of Pharmaceutics and Biopharmaceutics, Kiel University, 24118 Kiel, Germany; mtrenkel@pharmazie.uni-kiel.de

**Text Correction**

There was an error in the original publication [1]. The protocol of the slug mucosal irritation assay differed slightly from the protocol of the cited reference, and there was a mistake in Equation (2).

A correction has been made to Section 1, Introduction, Paragraph 5:

“However, the test was so far only used for liquid formulations, so the second aim of this study was to evaluate the SMI assay as predictive tool for nasal powders.”

should be replaced with:

“While the protocol described was evaluated with liquid formulations, mucus production from slugs was also used in other studies to assess the irritative potential of powders [22,23]. The second aim of this study was, therefore, to evaluate the irritative potential of different excipients for nasal powder formulations by using the SMIA.”

A correction has been made to Section 2.5, Evaluation of the Sensory Effects in the Nose, Equation (2):

(2) $$\mathrm{TM},\;\%=\overset{3}{\underset{i=1}{\sum }}M{\left(\mathrm{Mucus}\;\mathrm{per}\;\mathrm{CP},\;g\right)}_{i}/\;\mathrm{BW},\;g\;\times \;100\%$$

A correction has been made to Section 2.5, Evaluation of the Sensory Effects in the Nose, Paragraph 5:

“All of the experiments were conducted with three slugs that were not used in any experiments before.”

should be replaced with:

“Different from the protocol used by Lenoir et al. [21,31], the body weight of the slugs was not re-determined before each contact period, but the mucus production was related to the initial body weight before the first contact period.”

Two corrections have been made to Section 3.2, Evaluation of the Sensory Effects in the Nose, Paragraph 2:

“However, with the exception of mannitol with a small particle size, they are all within a range which is not associated with discomfort, according to [21].”

should be replaced with:

“However, compared to benzalkonium chloride as a marker for severe irritation, the mucus production was clearly reduced (*p* < 0.01), so we assume a minor irritation potential from the fillers.”

“The influence of particle size was evaluated for mannitol, and a significantly higher mucus production was found for the sieve fraction 32–90 µm than for the sieve fractions 32–150 µm or 90–150 µm. The obtained total mucus production of 6.30 ± 0.61% of the initial body weight of the slugs indicates mild irritation in the nose, possibly due to a faster dissolution of the smaller particles and hence a more pronounced osmotic effect. Comparing the mucoadhesive excipients, carboxymethyl chitosan stood out with a high total mucus production of 17.52 ± 0.63%, which is associated with severe nasal discomfort, and ranges only slightly below benzalkonium chloride (BAC) 1% solution as positive control.”

should be replaced with:

“The influence of particle size was evaluated for mannitol, and a significantly higher mucus production was found for the sieve fraction 32–90 µm than for the sieve fractions 32–150 µm or 90–150 µm, possibly due to a faster dissolution of the smaller particles and, hence, a more pronounced osmotic effect. Comparing the mucoadhesive excipients, carboxymethyl chitosan stood out, with a high total mucus production of 17.52 ± 0.63%, which ranges only slightly below benzalkonium chloride (BAC) 1% solution as a positive control.”

**Error in Figure**

In the original publication, there was a mistake in Figure 5 as published. Due to slight differences in the protocol of the slug mucosal irritation assay, the categories of nasal discomfort are not applicable. The corrected Figure 5 appears below. The figure legend has been corrected accordingly and appears below.

**Figure 5:**
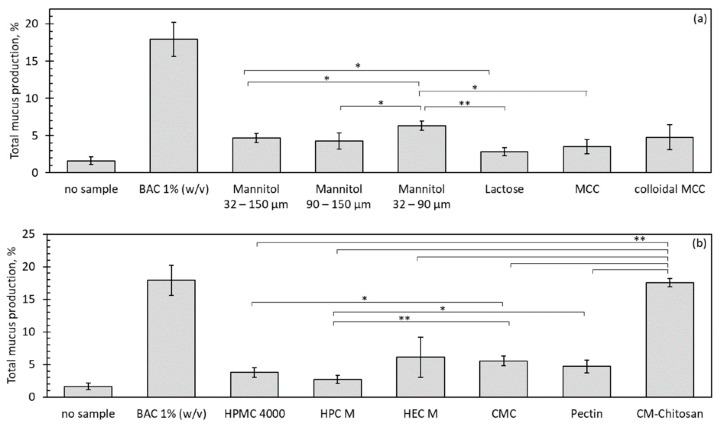
Total mucus production from three contact periods in the slug mucosal irritation assay, expressed as a percentage of the initial bodyweight of the slugs. (**a**) Fillers; (**b**) mucoadhesive polymers. Unless otherwise stated, sieve fractions from 32–150 µm were used. *n* = 3, error bars = sd, * = *p* < 0.05, ** = *p* < 0.01.

**References Added**

There are two newly added references [22] and [23], and the citations have been inserted to Section 1, Introduction, Paragraph 5. With this correction, the order of some references has been adjusted accordingly.

22. Callens, C.; Adriaens, E.; Dierckens, K.; Remon, J.P. Toxicological evaluation of a bioadhesive nasal powder containing a starch and Carbopol^®^ 974 P on rabbit nasal mucosa and slug mucosa. *J. Control. Release* **2001**, *76*, 81–91. https://doi.org/10.1016/S0168-3659(01)00419-9.
23. Adriaens, E.; Ameye, D.; Dhondt, M.M.M.; Foreman, P.; Remon, J.P. Evaluation of the mucosal irritation potency of co-spray dried Amioca^®^/poly(acrylic acid) and Amioca^®^/Carbopol^®^ 974P mixtures. *J. Control. Release* **2003**, *88*, 393–399. https://doi.org/10.1016/S0168-3659(03)00012-9.

The authors state that the scientific conclusions are unaffected. This correction was approved by the Academic Editor. The original publication has also been updated.

## References

[1] Trenkel M., Scherließ R. (2021). Nasal Powder Formulations: In-Vitro Characterisation of the Impact of Powders on Nasal Residence Time and Sensory Effects. Pharmaceutics.

